# Supplementary material for: Bridging the gap between sustainability and profitability: unveiling the untapped potential of sea cucumber viscera
Source: PeerJ. 2023 Oct 11;11:e16252. doi: 10.7717/peerj.16252 (PMC10576502; doi:10.7717/peerj.16252)
Supplement: Supplemental Information 1 [file peerj-11-16252-s001.docx]

Supplementary Table 1. The Distribution of Common Sea Cucumbers.

| **Species** | **Distribution** | **References** |
| --- | --- | --- |
| *Actinopyga lecanora* | Asia and East Africa, Indian Ocean, Indo-west Pacific | Ghanbari et al. (2012), Zhong et al. (2021) |
| *Actinopyga echinites* | Indian Ocean, tropical Pacific, southern Japan | Clark and Rowe (1971), Wiedemeyer (1994), Kohler et al. (2009) |
| *Actinopyga mauritiana* | Indian Ocean, western Pacific Ocean, Mariana Island, | Skewes et al. (2010), Trianni and Bryan (2004) |
| *Bohadschia argus* | Western Indian Ocean, Pacific Ocean | Purcell et al. (2016b), Woo et al. (2013). |
| *Bohadschia marmorata* | Indo-Pacific, North-East coastal region of Sri Lanka | Woo et al. (2013), Veronika et al. (2018). |
| *Cucumaria frondosa* | Indo-West Pacific, and Northeast coast of Scotland, Shetland and Orkney, West Atlantic, North Atlantic Ocean, Barents Sea (Russia) | Bruckner et al, (2003),  Zulfigar et al. (2008), Hamel et al. (1996), Hossain et al. (2020) |
| *Colochirus robustus* | Central Indo-Pacific, Eastern Indian | Woo et al, 2013 |
| *Euapta godeffroyi* | Tropical Indo-Pacific, Red Sea, Madagascar to Hawaii, northern Australia | Cannon and Silver (1986), Woo et al. (2013) |
| *Holothuria hermanni* | Indo-Pacific, Tropical West Atlantic, Tropical Australia, Mozambique, Indian Ocean, North Arabian Sea and Pacific coast of Central America. | Clark and Rowe (1971), Mosher (1980), Siddique and Ayub (2015) |
| *Holothuria leucospilota* | East coast of Africa, Indo-Pacific, North East coast of Australia | Yu et al. (2013), Woo et al. (2013). |
| *Holothuria atra* | Indo-Pacific from Red Sea and East Africa to Australia, Thailand, Eastern Pacific, Central Maluku | Bruckner et al. (2003),  Clark & Rowe (1971), Woo et al. (2013), Viyakarn et al. (2020), Ongkers et al. (2018). |
| *Holothuria cinerascens* | Pacific and Indian Oceans from Red Sea to Madagascar and Japan to Australia, Indo-Pacific, South China Sea, Red Sea to Hawaii | Clark & Rowe (1971), Massin (1996) |
| *Holothuria edulis* | Tropical Indo-Pacific Ocean, East African coast, Northern Australia, and various Pacific Island | Bruckner et al. (2003),  Woo et al. (2013), Dissanayake and Athukorala (2011) |
| *Holothuria fuscogilva* | Tropical Indo-Pacific Ocean, South-Pacific, Indian Ocean, Southeast Asia | Reichenbach (1999), Bruckner et al. (2003) |
| *Holothuria hilla* | Tropical Indo-West Pacific, Central Pacific, Southeast Asia, east Africa, Indian Ocean, South-Pacific, South China Sea, Persian Gulf | Clark & Rowe (1971), Purcell et al. (2012) |
| *Holothuria impatiens* | Tropical Indo-Pacific, Tropical Western Atlantic Ocean, Caribbean Sea, Gulf of Mexico, Mediterranean Sea and Coasts of France, Persian Gulf | Bruckner et al. (2003),  Clark & Rowe (1971), Afkhami et al. (2012), Dolorosa et al. (2017). |
| *Holothuria nobilis* | Indo-Pacific Ocean, Red Sea and Madagascar to Hawaii, Japan to Northern Australia, Indian Ocean, east coast of Africa, Arabian Seas. | Clark & Rowe (1971), Massin (1996), Stout (2021). |
| *Holothuria pardalis* | Indo-Pacific and Eastern Pacific, Red Sea to Hawaii, Pakistan, Indian Ocean | Clark & Rowe (1971), Cannon and Silver (1986), Ahmed et al. (2020) |
| *Holothuria pevircax* | Indo-West Pacific, Africa and Hawaii, Iran, Northern Oman Sea, Japan, Korea, India | Clark & Rowe (1971), Amiri et al. (2013), Yamada et al. (2002), Lee and Shin (2019), Idressbabu and Sureshkumar (2017) |
| *Holothuria scabra* | Indo-Pacific region from east Africa to the eastern Pacific, Iran, southwestern Pacific Ocean, Oman, South Pacific, Australia, China, India, Indonesia, Japan, Micronesia, Malaysia, Mozambique, Philippines, Thailand | Bruckner et al. (2003),  Clark & Rowe (1971), Mercier et al. (2000), Al-Rashdi et al. (2007), Hamel et al. (2001) |
| *Holothuria tubulosa* | North-eastern Atlantic Ocean, Mediterranean Sea, Algerian coast, Turkey, Greece, Italy, Spain | Rakaj et al. (2017), Mezali et al. (2021), Sicuro and Levine (2011). |
| *Paracaudina australis* | Australia, Queensland, South-East Coast, Southeast Asia, New Zealand | Widianingsih et al. (2019),  O’loughlin et al. (2011), Lane and Vandenspiegel (2003). |
| *Pearsonothuria graeffei* | Tropical Indo-Pacific Ocean from east coast of Africa to the Philippines, Indonesia, Taiwan, Madagascar, Maldives, Guam, Fiji and the South Pacific. | Woo et al. (2013), Dong et al. (2008) |
| *Stichopus hermanni* | West Indo-Pacific, Southeast Asia, and South-Pacific, Australia, Persian Gulf | Bruckner et al. (2003),  Clark & Rowe (1971), Khazaali et al. (2016). |
| *Stichopus japonicus* | Coast of Russia, China, Japan, Korea, Alaska, coastal waters of northeast Asia | Bruckner et al. (2003),  Yamana et al. (2009), Minami et al. (2018), Oh et al. (2017). |
| *Stichopus vastus* | Tropical and Western Indo-Pacific, Indian Ocean, Andaman Island, South China Sea, Indonesia, Thailand, Australia, Micronesia, Papua New Guine. | Clark and Rowe (1971), Massin et al. (2002), Abedin et al. (2012), Woo et al. (2013) |
| *Stichopus chloronotus* | Indo-West Pacific, Eastern Africa to Hawaii, South Pacific | Chen et al., 2021. |
| *Synaptula lamperti* | Western Pacific including the coast of Indonesia, Papua New Guinea, Micronesia, Philippines, Sri Lanka | Herath et al. (2020), Woo et al. (2013) |
| *Synapta maculate* | Indo-Pacific Ocean, South China Sea | Canada et al. (2020), Woo et al. (2013) |
| *Thelenota ananas* | Tropical Indo-Pacific from Red Sea and East Africa to Hawaii and Polynesia, Western Indian Ocean, South China Sea | Lane (1999), Eriksson et al. (2012), Woo et al. (2013), Liu and Shen, (2021). |
| *Thelenota anax* | Indo-West Pacific from Eastern Africa and Madagascar and the Comoros Island, east to Australia, Indonesia, Philippines, north Japan, | Clark and Rowe (1971), Woo et al. (2013), Conand et al. (2013). |
